# Supplementary material for: TRUE-1: Trial of Repurposed Unithiol for snakebite Envenoming phase 1 (safety, tolerability, pharmacokinetics and pharmacodynamics in healthy Kenyan adults)
Source: Wellcome Open Res. 2022 Mar 14;7:90. [Version 1] doi: 10.12688/wellcomeopenres.17682.1 (PMC8961198; doi:10.12688/wellcomeopenres.17682.1)
Supplement: Peer review history

# Peer review: Reviewer response for version 1

**DOI:** 10.21956/wellcomeopenres.19564.r49206

**Published:** 2022-03-28

**Authors:** Matthew Lewin

**Affiliations:** 1 California Academy of Sciences, San Francisco, CA, USA

The paper is well written. I don't see any major issue to be addressed. It is a very important step towards the modernization of snakebite envenoming treatment. The investigators are taking a disciplined, safety oriented, approach to evaluating a drug that has been around for some time. They are not taking any shortcuts. 

**Very minor comments/questions:**

1. It is possibly worth mentioning the safety of metal chelation therapies in patients with sickle cell disease/trait patients. This has been studied. There are no major issues I am aware of (studies seem to indicate good safety) but seems worth noting. 
2. CIV should be defined in the table subtext (I had to go back to the text to remind myself) and could consider a hyphen. CIV stands for a lot of things other than "cohort IV"...chemical injection valve, but more importantly "continuous IV infusion". Some people scan or read tables first to get a generic view of a paper before reading--or go back to the tables after reading but don't remember the abbreviations. Truly minor comment. 

**Two more observations - neither are major:**

1. It is not immediately clear to me exactly how you will decide if it is 2 days vs. 3 days. Also, I am looking at the 3 ^rd^ paragraph of the section titled, "Justification for the proposed dose, route of administration, dosage regimen and treatment period," and I don't see a clear justification for 2-3 days vs. a longer period of treatment. Maybe it's there, I just don't see it clearly explained.
2. The organization of Table 2 is helpful but there is a part I don't get. The last row is the single IV dose of 5-10 mg/kg. Not sure why this goes in the Multiple Dose section of the table. You do say (and CIV2) in the subheading but it was not easy for me as an outside reader to organize the groups. Why not just make it a separate bubble with a simple explanation (e.g. If x then y)? 

Is the study design appropriate for the research question?

Yes

Is the rationale for, and objectives of, the study clearly described?

Yes

Are sufficient details of the methods provided to allow replication by others?

Yes

Are the datasets clearly presented in a useable and accessible format?

Yes

Reviewer Expertise:

Basic research, clinical medicine

I confirm that I have read this submission and believe that I have an appropriate level of expertise to confirm that it is of an acceptable scientific standard.

# Peer review: Reviewer response for version 1

**DOI:** 10.21956/wellcomeopenres.19564.r49208

**Published:** 2022-03-28

**Authors:** Eric Lavonas

**Affiliations:** 1 Department of Emergency Medicine, Denver Health, Denver, CO, USA; 2 Rocky Mountain Poison and Drug Safety, Denver Health, Denver, CO, USA; 3 Department of Emergency Medicine, University of Colorado School of Medicine, Aurora, CO, USA

This is a thorough, clear, well-thought through clinical trial protocol. My suggestions are small and, I think, completely optional.

- Re: Timing of follow-up safety assessments: I think an additional assessment at 7 and/or 14 days would enhance safety. However, given the short half-life of unithiol, I see no need to have a follow up beyond the 42-day assessment. The 6-month assessment is likely to find a log of unrelated health events with little to no chance of finding clinically relevant safety information.
- Re: Exclusion criteria. I agree it is appropriate to exclude children, the elderly, and patients with asthma in this phase 1 trial. However, these patient populations do suffer snake envenoming. Can we comment on a plan to include them in future research, if unithiol proves safe in the current trial population?
- Re: Lab testing: I suggest also measuring serum/plasma concentrations of calcium, magnesium, and zinc.
- Re: Lab testing. I understand that unithiol is not metabolized by the CYP450 enzyme system. However, I don't think the current data exclude the possibility of a clinically important minor metabolite from one/more of these pathways. Suggest you obtain and hold blood for CYP450 and other relevant genotyping. That way, if a patient has a SAE, you have the samples you need to do genotyping in a case-control fashion and understand what happened.
- Re: Stability of lab samples: I do have some concern that unithiol / metabolites may be unstable in stored serum / plasma samples, even if frozen. Degradation during storage and transport to the UK and/or artifact from the freezing and storing process is a major threat to the study. I suggest you obtain multiple serum / plasma tubes at a few time points, run duplicate analyses, and measure the variance/reliability of your assay results. Of course, it would be better to run samples on-site, but I understand this might not be possible.
- Re: Multiple dosing: Please clarify dosing frequency in the multiple dosing arm. Are subjects receiving unithiol daily, or (as suggested on page 9, column 1, bottom) 4 times a day?
- Re: No placebo group. While I think this is ethical and perfectly OK for a phase 1 trial, please consider that there are known changes in liver function that occur when subjects are admitted to a clinical trials unit. These are likely related to diet and exercise (subjects eat more and exercise less when housed in a CTU). The changes can be hard to distinguish from early signs of liver injury. I apologize that I can't find the supporting reference for this, and it may be a non-issue in such a short protocol, but the trial would be cleaner if it included at least a few placebo subjects. Interestingly, there also appear to be systematic changes in liver test results placebo-group subjects in non-residential trials (see: https://journals.physiology.org/doi/full/10.1152/ajpgi.00051.2018). ^1^ 
- Re: Vital signs: Axillary temperature is quite inaccurate. I suggest you use oral temperature. If there's a reason oral is not appropriate, then most studies show temporal artery temperature measurements are more accurate than axillary.
- Re: Follow up on infants born to exposed mothers: The plan as described is standard and probably adequate given the safeguards in place in this study, but it's worth noting that even major neurodevelopmental outcomes may not be apparent at 6 months of age. Additional assessments (even telephonic) at 12 and 24 months would improve safety surveillance for infants born to mothers in this study. Again, I think the risk is low, safety plans are thorough, and women who are or could become pregnant are a relevant population to snake envenoming, so I am not criticizing the plan.
- Re: PK calculations: Before calculating the elimination half-life, it is worth assessing the elimination for fit. With enough subjects and sampling, you may find mixed kinetics.
- Re: Elimination: Should the study also assess for the fate of metabolites?
- Re: Pharmacodynamic outputs: This is an area I know very little about, and can provide no expertise. Please ensure the protocol is reviewed by someone with a good working familiarity with the methods proposed here. 

Thank you for inviting me to review this excellent protocol. The work proposed is important and will be useful to benefit patients with snake envenoming.

Is the study design appropriate for the research question?

Yes

Is the rationale for, and objectives of, the study clearly described?

Yes

Are sufficient details of the methods provided to allow replication by others?

Yes

Are the datasets clearly presented in a useable and accessible format?

Yes

Reviewer Expertise:

Snake envenomation, clinical trials, resuscitation

I confirm that I have read this submission and believe that I have an appropriate level of expertise to confirm that it is of an acceptable scientific standard.

## References

[1] : Factors influencing longitudinal changes of circulating liver enzyme concentrations in subjects randomized to placebo in four clinical trials. *American Journal of Physiology-Gastrointestinal and Liver Physiology* .2019;316(3) : 10.1152/ajpgi.00051.2018 G372-G386 10.1152/ajpgi.00051.2018 30495974
